# RETRACTION: A Meta‐Analysis Examined the Effect of Topical Nursing Application of Antimicrobial as a Prophylaxis for the Stoppage of Surgical Wound Infection in Colorectal Surgery

**DOI:** 10.1111/iwj.70506

**Published:** 2025-04-18

## Abstract

**RETRACTION:** WangX. , FuL. , GuoS. , and FangX. , “A Meta‐Analysis Examined the Effect of Topical Nursing Application of Antimicrobial as a Prophylaxis for the Stoppage of Surgical Wound Infection in Colorectal Surgery,” International Wound Journal 20, no. 6 (2023): 2010–2019, 10.1111/iwj.14064.36727574 PMC10333037

The above article, published online on 02 February 2023, in Wiley Online Library (http://onlinelibrary.wiley.com/), has been retracted by agreement between the journal Editor in Chief, Professor Keith Harding; and John Wiley & Sons Ltd. Following an investigation by the publisher, all parties have concluded that this article was accepted solely on the basis of a compromised peer review process. In addition, the investigation found significant textual overlap between this article and another article by different authors [1]. The editors have therefore decided to retract the article. The authors did not respond to our notice regarding the retraction.

Reference

[1] NelsonR. L. , KravetsA. , KhateebR. , RazaM. , SiddiquiM. , TahaI. , TummalaA. , EppleR. , HuangS. , and WenM. , “Topical Antimicrobial Prophylaxis in Colorectal Surgery for the Prevention of Surgical Wound Infection: a Systematic Review and Meta‐Analysis,” Techniques in Coloproctology 22 (2018): 573–587, 10.1007/s10151-018-1814-1.30019145

**RETRACTION:** X. Wang , L. Fu , S. Guo , and X. Fang , “A Meta‐Analysis Examined the Effect of Topical Nursing Application of Antimicrobial as a Prophylaxis for the Stoppage of Surgical Wound Infection in Colorectal Surgery,” International Wound Journal 20, no. 6 (2023): 2010–2019, 10.1111/iwj.14064.36727574 PMC10333037

The above article, published online on 02 February 2023, in Wiley Online Library (http://onlinelibrary.wiley.com/), has been retracted by agreement between the journal Editor in Chief, Professor Keith Harding; and John Wiley & Sons Ltd. Following an investigation by the publisher, all parties have concluded that this article was accepted solely on the basis of a compromised peer review process. In addition, the investigation found significant textual overlap between this article and another article by different authors [1]. The editors have therefore decided to retract the article. The authors did not respond to our notice regarding the retraction.

**Reference**

[1] R. L. Nelson , A. Kravets , R. Khateeb , M. Raza , M. Siddiqui , I. Taha , A. Tummala , R. Epple , S. Huang , and M. Wen , “Topical Antimicrobial Prophylaxis in Colorectal Surgery for the Prevention of Surgical Wound Infection: a Systematic Review and Meta‐Analysis,” Techniques in Coloproctology 22 (2018): 573–587, 10.1007/s10151-018-1814-1.30019145

